# The Effect of Splenectomy on the Reversal of Cirrhosis: a Prospective Study

**DOI:** 10.1155/2019/5459427

**Published:** 2019-04-08

**Authors:** Dao-Bing Zeng, Liang Di, Rui-Chi Zhang, Qing-Liang Guo, Bin-Wei Duan, Cui-Yu Jia, Feng Chen, Dong-Dong Lin, Yun-jin Zang, Shi-Chun Lu

**Affiliations:** ^1^General Surgery Department, Beijing You'an Hospital, Capital Medical University, Beijing 100069, China; ^2^Department of Radiology, Beijing You'an Hospital, Capital Medical University, Beijing 100069, China; ^3^Institute & Hospital of Hepatobiliary Surgery of Chinese PLA, Chinese PLA Medical School, Chinese PLA General Hospital, Beijing 100853, China

## Abstract

**Background:**

Studies have demonstrated that liver fibrosis can be reversed by medication treatments. After splenectomy, cirrhosis patients have short-term changes in several serum markers for cirrhosis and liver stiffness.

**Aims:**

To investigate the effect of splenectomy on the severity of cirrhosis.

**Methods:**

A total of 62 patients with cirrhosis and portal hypertension receiving splenectomy from December 2014 to July 2017 were enrolled. The degree of cirrhosis was preoperatively and postoperatively evaluated by serum markers, including hyaluronan (HA), laminin, amino-terminal propeptide of type III procollagen (PIIINP), type IV collagen (C-IV), liver stiffness (FibroScan), and liver volume.

**Results:**

HA levels significantly increased at 1 week and 1 month postoperation (both *P* < 0.05), whereas the levels of PIIINP and C-IV significantly decreased from 1 month to 12 months postoperation (all *P* < 0.05). In addition, elastography examination demonstrated that the FibroScan score significantly reduced from 1 month to 24 months postoperation as compared with the baseline level (all *P* < 0.05). CT scan showed that the liver volume significantly increased at 6 months postoperation (*P* < 0.05). Furthermore, the alteration trends of these serum markers and the FibroScan score were further confirmed by the multivariate linear regression.

**Conclusions:**

These observations suggested that splenectomy may result in long-term reversal of cirrhosis.

## 1. Introduction

Cirrhosis is a pathological state of the liver characterized by fibrosis and morphologic conversion of normal liver tissue into abnormal nodules, which is the final stage of various chronic hepatic disorders [1]. There are a variety of causes of liver cirrhosis, including viral, alcoholic, autoimmune alcoholic, and fatty liver diseases [1]. Cirrhosis was traditionally considered as an irreversible disease. Nevertheless, in the past decade, both preclinical [2, 3] and clinical studies [4–8] have provided evidence demonstrating that liver fibrosis can be reversed to some extent, and even cirrhosis can be histologically reversed. For example, Chang et al. have demonstrated that long-term entecavir therapy in patients with chronic hepatitis B induces substantial histological improvement and regression of fibrosis or cirrhosis [8]. Kim et al. have reported that candesartan (an angiotensin-blocking agent) treatment results in significant improvement of fibrosis in histological and quantitative assessments in alcoholic hepatitis [6]. A retrospective study on (*n* = 87) by Czaja and Carpenter have revealed that corticosteroid therapy improves fibrosis in 53% of patients with autoimmune hepatitis [5]. All these observations strongly suggest that medication treatments may have the beneficial effect of reversal of cirrhosis.

The current “gold standard” for evaluating the degree of liver fibrosis or cirrhosis remains ultrasound-guided liver biopsy. However, liver biopsy is invasive and may induce complications such as pain, pneumothorax, hemorrhage, and perforation, which limit its clinical application [9]. In addition, sampling bias and variability may lead to inaccurate staging in liver biopsy [10]. Currently, several noninvasive diagnostic tools have been developed for the assessment of liver fibrosis, including serum markers, imaging examinations, and liver stiffness measurements [11, 12]. Serum cirrhosis markers include hyaluronan (HA) [13], laminin (LN), amino-terminal propeptide of type III procollagen (PIIINP) [14], type IV collagen (C-IV) [15], matrix metalloproteinases (MMPs) [16], tissue inhibitor of metalloproteinases-1 (TIMP-1) [17], and aspartate aminotransferase to alanine aminotransferase ratio (AST/ALT) [18]. Imaging diagnostic methods for liver cirrhosis include abdominal ultrasonography (US), computed tomography (CT), and magnetic resonance imaging (MRI) [19]. Transient elastography, such as FibroScan, is a method developed for measurement of liver stiffness and diagnosis of fibrosis and cirrhosis [20].

Our previous study found that after cirrhosis patients receiving splenectomy, there are short-term changes in several serum markers for cirrhosis and liver stiffness (FibroScan value) [21], indicating that splenectomy has a short-term effect on serum fibrosis markers and liver stiffness in cirrhosis patients. This phenomenon raises the possibility that splenectomy might be able to induce reversal of cirrhosis. To verify this hypothesis, this prospective study is aimed at investigating the long-term effect of splenectomy on the degree of cirrhosis by using serum markers, imaging examinations, and liver stiffness measurement.

## 2. Materials and Methods

### 2.1. Participants

A total of 62 patients with cirrhosis and portal hypertension who underwent splenectomy with/without esophagogastric devascularization in our hospital from December 2014 to July 2017 were enrolled in this prospective study. The inclusion criteria were (1) age of 20-65 years, (2) clinically or pathologically confirmed cirrhosis and portal hypertension (including viral hepatitis, alcoholic hepatitis, and autoimmune hepatitis), (3) diameter of splenic artery > 5.19 mm or the ratio of splenic artery diameter to proper hepatic artery diameter > 1.4, and (4) preoperative evaluation which showed stable vital signs, Child-Pugh grades A and B; patients can tolerate the abdominal surgery under general anesthesia. The exclusion criteria are (1) idiopathic portal hypertension, (2) Budd-Chiari syndrome, and (3) poor compliance, preoperative evaluation which showed vital signs instability, need to use vasoactive drugs to maintain blood pressure, severe hepatic encephalopathy symptoms, and severe coagulation dysfunction. This study was approved by the institutional review board of the Beijing You'an Hospital, Capital Medical University. Written informed consent was obtained from each patient.

### 2.2. Surgical Management

Patients with a history of upper gastrointestinal bleeding (*n* = 47) underwent splenectomy combined with esophagogastric devascularization as previously described [22]. For patients without a history of upper gastrointestinal bleeding (*n* = 15), after splenectomy, it was determined whether esophagogastric devascularization should be carried out based on the intraoperative portal pressure [23]. Five patients did not receive esophagogastric devascularization because the portal pressure had been reduced to normal level after splenectomy. Two patients combined with hepatocellular carcinoma simultaneously underwent partial hepatectomy. All hepatitis B patients (*n* = 47) received antiviral therapy before and after surgery.

### 2.3. Determining the Serum Markers of Cirrhosis

For evaluating the degree of cirrhosis, the serum markers (HA, LN, PIIINP, and C-IV) were determined. At preoperation, 1 week, 1 month, 3 months, 6 months, and 12 months postoperation, peripheral blood samples were collected and the levels of the above serum markers were determined using the sensitized chemiluminescence immunoassay detection system (JETLIA 96/2; China Medical Technologies, Beijing, China) on the same day.

### 2.4. Assessment of Liver Stiffness by FibroScan

Liver stiffness measurement was performed at preoperation, 1 month, 3 months, 6 months, 12 months, and 24 months postoperation using continuous FibroScan (Echosens, Paris, France) according to the manufacturer's protocol. Briefly, the patient was placed in the supine position, and the scanning was conducted in the region encompassing the 7th, 8th, and 9th intercostal spaces between the anterior axillary and midaxillary lines. The number of successful detections should be larger than 10, and the median value of FibroScan value (expressed in kPa) was recorded as the final FibroScan score. In addition, the interquartile range/median FibroScan score should be smaller than 33% and the detection success rate should be greater than 60%. The ultrasound was performed using the Aixplorer diagnostic ultrasound system (Supersonic Imagine, France).

### 2.5. Evaluation of Liver Volume

Imaging examinations were conducted at preoperation, 1 month, 3 months, 6 months, 12 months, and 24 months postoperation. Liver volume scanning was conducted using a LightSpeed VCT 64-slice CT scanner (GE. Healthcare, USA). The image was acquired using a three-phase enhanced scanner (arterial phase 20-25 s, portal venous phase 65-70 s, and equilibrium phase 180 s), with a scanning range from the dome of the diaphragm to the lower edge of the liver and spleen. A nonionic contrast medium was injected into the elbow vein using a high-pressure syringe. After image acquisition, the data was used for liver volume reconstruction and assessment using the Advantage Workstation 4.3 software (GE Healthcare) according to the manufacturer's protocol.

### 2.6. Statistical Analysis

Continuous data were expressed as mean ± standard deviation (SD) and compared by Student's paired *t*-test. If normality was not assumed, the Wilcoxon sum-of-rank test would be used for comparisons between dependent variables. Categorical data were indicated by number and percentage (%). One-way repeated measurement ANOVA was used for the comparisons among time points (from preoperation to 2 years), and using Fisher's LSD comparisons for the post hoc test. Univariate and multivariate generalized estimating equation (GEE) and linear regression models were used to investigate the change among time points to the results of markers (HA, LN, PIIINP, C-IV, FibroScan, and liver volume). A first-order autoregressive working correlation matrix was adopted for the repeated measures data. Patients' age, sex, and Child-Pugh score were controlled as covariates in multivariate models. The significant level of all analyses was set at a *P* value <0.05, two-tailed. All analyses were performed using IBM SPSS Version 20 (SPSS Statistics V20, IBM Corporation, Somers, New York, USA).

## 3. Results

### 3.1. Patients' Clinical Features

A total of 62 eligible patients (mean age = 46.90 ± 9.58 years) were enrolled in this study, including 34 (54.84%) male and 28 (45.16%) female. Patients' demographic and clinical characteristics, as well as the details of the operation, are summarized in Table 1. Hepatitis B virus (HBV) infection (*n* = 47, 75.81%) was the most common cause of cirrhosis. Other causes of cirrhosis included hepatitis C virus (HBC) infection (*n* = 9, 14.52%), alcoholic fatty liver (*n* = 10, 16.13%), primary biliary cirrhosis (PBC, *n* = 3, 4.84%), primary sclerosing cholangitis (PSC, *n* = 1, 1.61%), and autoimmune hepatitis (AIH, *n* = 2, 3.23%). The mean surgical time was 214.25 ± 49.28 min. The amounts of operative bleeding, transfusion of red blood cells, and plasma were 201.64 ± 187.56 mL, 91.80 ± 257.10 mL, and 170.49 ± 287.14 mL, respectively. Following splenectomy, 4 patients developed postoperative intraperitoneal hemorrhage, which was resolved by emergency exploratory laparotomy to stop bleeding (*n* = 3) or conservative drug treatment (*n* = 1). One case had upper gastrointestinal bleeding at 8 days after operation, which were resolved by drug therapy for hemostasis and portal hypertension. One patient with pancreatic leakage (grade I) was treated with conservative treatment. One case with abdominal infection (*Staphylococcus epidermidis*) was treated with tienam and vancomycin.

### 3.2. The Change of Serum Markers, FibroScan Score, and Liver Volume

**Figure 1:**
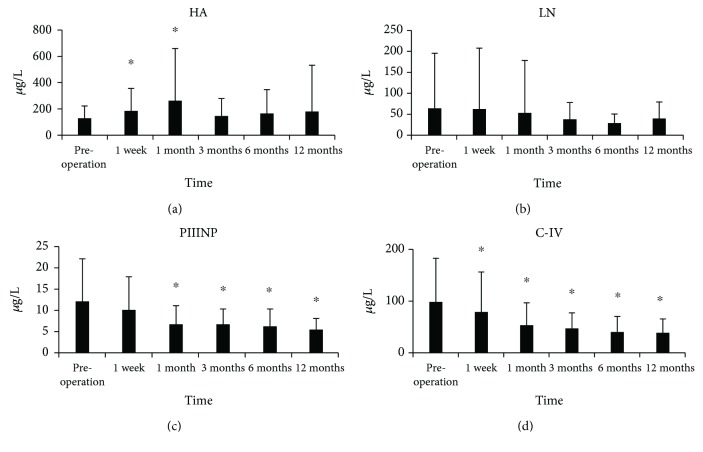
The changes in patients' serum markers, including HA (a), LN (b), PIIINP (c), and C-IV (d). ^∗^*P* < 0.05 compared to preoperation. The numbers of compared pairs of all serum markers at 1 week, 1 month, 3 months, 6 months, and 12 months were 48, 39, 30, 27, and 15, respectively. The sample sizes of HA were 51, 50, 40, 32, 29, and 16 to preoperation, 1 week, 1 month, 3 months, 6 months, and 12 months, respectively. The sample sizes of LN were 51, 50, 40, 32, 29, and 16 to preoperation, 1 week, 1 month, 3 months, 6 months, and 12 months, respectively. The sample sizes of PIIINP were 51, 50, 40, 32, 29, and 16 to preoperation, 1 week, 1 month, 3 months, 6 months, and 12 months, respectively. The sample sizes of C-IV were 51, 50, 40, 32, 29, and 16 to preoperation, 1 week, 1 month, 3 months, 6 months, and 12 months, respectively.

The median follow-up time was 12 months (range: 1-24 months). Five patients did not return to our hospital for follow-up due to living in remote areas. During the follow-up, one patient died of an accidental fall, and one patient died of multiple autoimmune diseases at 16 months postoperation. One patient was diagnosed with HCC at one year postoperation and was withdrawn from the study. After splenectomy, the patient's portal vein pressure was significantly decreased (34.15 ± 5.03 vs. 25.70 ± 4.19, *P* < 0.001). In addition, platelet counts significantly increased at all the time points after splenectomy (Table 2, all *P* < 0.05). In addition, the Child-Pugh score was significantly decreased at 1, 3, 6, 12, and 24 months after splenectomy (Table 2, all *P* < 0.05). To investigate if splenectomy has an effect on the degree of cirrhosis, the serum markers of cirrhosis (HA, LN, PIIINP, and C-IV) were determined. Compared to the corresponding baseline (preoperation) levels, HA was significantly increased at 1 week and 1 month postoperation (Figure 1(a), both *P* < 0.05). However, no significance was found in LN (Figure 1(b), all *P* > 0.05). PIINP was significantly decreased from 1 month to 12 months postoperation (Figure 1(c), all *P* < 0.05), and C-IV was significantly reduced from 1 week to 12 months postoperation (Figure 1(d), all *P* < 0.05).

**Figure 2:**
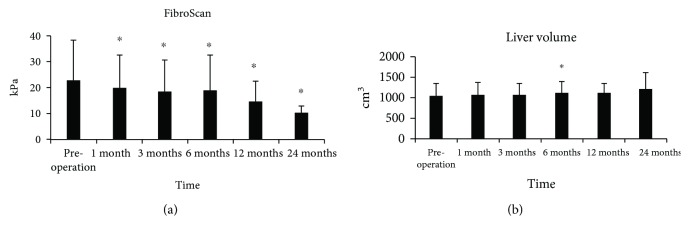
The change of patients' FibroScan (a) and liver volume (b). ^∗^*P* < 0.05 compared to preoperation. The numbers of compared pairs of FibroScan at 1 month, 3 months, 6 months, 12 months, and 24 months were 45, 37, 30, 31, and 6, respectively. The numbers of compared pairs of liver volume at 1 month, 3 months, 6 months, 12 months, and 24 months were 37, 28, 28, 22, and 5, respectively. The sample sizes of FibroScan were 55, 48, 40, 34, 32, and 6 to preoperation, 1 month, 3 months, 6 months, 12 months, and 24 months, respectively. The sample sizes of liver volume were 50, 44, 36, 35, 30, and 6 to preoperation, 1 month, 3 months, 6 months, 12 months, and 24 months, respectively.

Meanwhile, elastography examination demonstrated that the FibroScan score was significantly reduced from 1 month to 24 months postoperation as compared with the baseline level (all *P* < 0.05, Figure 2(a)). CT scan revealed that the liver volume was only significantly increased at 6 months postoperation (*P* < 0.05, Figure 2(b)).

### 3.3. Multivariate Linear Regression Results with GEE Models

To further confirm the change trends of the serum and imaging markers, the multivariate linear regression with GEE models adjusted for patients' sex, age, and Child-Pugh score was performed. As shown in Table 3, compared to their corresponding reference time points (preoperation), HA was significantly elevated at 1 week and 1 month (both *P* < 0.05), while PIINP and C-IV were significantly reduced from 1 month to 12 months (all *P* < 0.05). However, no significance was found in LN (all *P* > 0.05).

As shown in Table 4, the FibroScan score was significantly decreased from 1 month to 24 months postoperation as compared with the baseline (all *P* < 0.05), and the liver volume was only significantly increased at 24 months postoperation (*P* < 0.05).

## 4. Discussion

Even though previous studies have demonstrated that medication treatments can result in reversal of cirrhosis, however, studies on the effect of splenectomy on the degree of cirrhosis are limited [24]. In this study, we investigated the effect of splenectomy on the reversal of cirrhosis. The results showed that compared to the baseline level, HA levels significantly increased at 1 week and 1 month postoperation, whereas the levels of PIIINP and C-IV significantly decreased from 1 month to 12 months postoperation. In addition, the FibroScan score significantly reduced from 1 month to 24 months postoperation. CT scan showed that the liver volume significantly increased at 6 months postoperation. Furthermore, the alteration trends of these serum markers and FibroScan score were further confirmed by multivariate linear regression. Taken together, these observations suggested that splenectomy may result in reversal of cirrhosis.

Our previous study showed that the optimal cutoffs for abnormal splenic artery internal diameter and S/P ratio in cirrhosis-induced portal hypertension are >5.19 mm and >1.40, respectively, which could be a marker for splanchnic hemodynamic disturbances [25]. Therefore, only patients with a splenic artery internal diameter > 5.19 mm and S/P ratio > 1.40 were enrolled in this study. Liver fibrosis is a consequence of disorganization of extracellular matrix (ECM) components, which cause loss of normal liver cell function [26, 27]. In fibrotic liver, the ECM metabolites are significantly increased so that the serum levels of ECM components, such as HA, LN, PIIINP, and C-IV, can be used as markers for the stage and progression of cirrhosis [28]. For instance, HA reflects the liver fibrogenesis and liver injury [29], while PIIINP and C-IV indicate the metabolism of collagens [30]. The elevated serum HA level in the cirrhosis patient is due to the fact that the liver sinusoidal endothelial cells reduce the uptake of HA [31]. It has been shown that following partial hepatectomy, hepatic stellate cells synthesized large amounts of HA during liver regeneration [32], suggesting that liver regeneration is associated with elevated HA level. In this study, serum HA level was significantly elevated at 1 week and 1 month postoperation. Our previous study showed that serum HA level does not alter immediately postoperation, but significantly increases at 2 days and 1 week postoperation. Meanwhile, although the serum HA levels were slightly higher at 3, 6, and 12 months postoperation than at preoperation, the differences did not reach significance. These observations implied that reversal of cirrhosis might be started early at 2 days after splenectomy. However, further evidence is necessary to support this suggestion.

Collagens are synthesized by hepatic stellate cells as precursor molecules, followed by cleaving at both N- and C-terminal ends by proteinases, and the mature collagen is then integrated into the ECM. Hence, both the procollagen and the propeptide can reflect the synthesis of ECM [28]. PIIINP is a well-studied marker of liver fibrosis [33, 34]. It has been shown that PIIINP has a high sensitivity and specificity to detect cirrhosis [33]. C-IV plays important roles in the pathogenesis of fibrosis disease, and the serum levels of C-IV can be used for predicting the state of liver fibrosis [28]. In this study, the serum level of PIINP was significantly decreased from 1 month to 12 months after splenectomy, and the C-IV level was significantly reduced from 1 week to 12 months postoperation.

FibroScan (transient elastography) is a method for the assessment of liver fibrosis through measuring liver stiffness by a monodimensional ultrasound [35]. A meta-analysis by Shaheen et al. has reported an excellent diagnostic accuracy of FibroScan for HCV-related cirrhosis with an area under the curve of the receiver operating characteristic (AUROC) of 0.95 [36]. Moreover, FibroScan can provide better diagnostic performance for predicting liver fibrosis than serum markers [35]. In the current study, the preoperative FibroScan values of all patients were greater than 21 kPa, suggesting severe cirrhosis. After splenectomy, the FibroScan values exhibited a continuous decrease trend, and all the postoperative FibroScan values were significantly lower than the preoperative ones. At 24 months postoperation, the FibroScan values could be reduced to 10 kPa. Taken together, our serum markers and liver stiffness measurement suggested that after splenectomy, reversal of cirrhosis might be started early at 1 week and can last for at least 2 years. To our best knowledge, this is the first study reporting the long-term effect of splenectomy on the reversal of cirrhosis. However, it is worth to further elucidate the mechanism underlying splenectomy-induced reversal of cirrhosis. It should be pointed out that the reduced portal hypertension following splenectomy may also decrease the liver stiffness.

Accumulating evidence has suggested that there is a significant volume reduction in cirrhotic livers as compared with normal livers [37, 38]. In addition, long-term oral nucleos(t)ide analogue therapy in patients with HBV-related liver compensated and decompensated cirrhosis leads to a significant increase in liver volume [39]. In this study, we found that although the liver volume was increased at all the time points after splenectomy, only the difference at 6 months postoperation reaches statistical significance, which may be attributed to the small sample size of this study. A study with a large sample size should be conducted to further validate this issue.

Liver fibrosis frequently causes portal hypertension [40], in turn leading to hypersplenism [41]. *Portal hypertension-induced* hypersplenism causes thrombocytopenia [41]. Splenectomy can effectively reduce portal pressure and correct hypersplenism and improve hypersplenism-induced thrombocytopenia [42, 43]. In this study, patients' portal vein pressure was significantly decreased after splenectomy. In addition, platelet counts significantly increased at all the time points after splenectomy. Since the progression of fibrosis parallels the increase in portal pressure [44], it is worth investigating if reduction in the portal pressure after splenectomy contributes to the reversal of cirrhosis.

In addition to splenectomy, several nonsurgical methods have been utilized to treat hypersplenism. Microwave ablation can improve hypersplenism in cirrhosis patients, but multiple ablations are required, with mean ablation times of 8.8 ± 1.3, and the therapeutic effect is not as good as splenectomy [45]. Although high-intensity focused ultrasound improved hypersplenism, both white blood cells (WBCs) and platelet counts did not return to normal levels [46]. Studies have shown that in children with chronic liver disease with hypersplenism [47] and cirrhosis in adults [48], after propranolol treatment for 1-4 weeks, platelet counts significantly increase. However, the follow-up durations on these two studies are both short, and their long-term efficacy is uncertain. Although splenic artery embolization can improve WBCs and platelet in patients with hypersplenism, it may cause severe complications [49]. The incidence of the postembolization syndrome is high to 77.8-100% [50]. The incidence of morbidity and complications after splenic embolization was higher than that of splenectomy, and the 1-year effective rate was only 16% [51]. Radiotherapy for hypersplenism can only increase the platelet counts but not increase WBCs and red blood cells (RBCs) [52]. By contrast, in a study of 226 patients with splenectomy and followed up for 3-96 months (mean = 63), splenectomy effectively increased WBCs, platelet counts, and RBCs [53], indicating that splenectomy improves spleen function with long-term efficacy.

Several limitations of the current study should be pointed out. First, we only adopted noninvasive methods to assess the degree of cirrhosis. The improvement in markers of fibrosis and liver stiffness may be attributed to the reduced portal hypertension following splenectomy, and reversal of cirrhosis should be further supported by pathological evidence. Nevertheless, the pathological examination cannot be repeatedly carried out in a short time due to its invasiveness and complications. Hence, we chose to adopt serum markers and liver stiffness measurement for assessment of the degree of cirrhosis in this trial. In addition, the sample size of this study was relatively small, and two patients having undergone hepatectomy may cause heterogeneity of the enrolled population. Furthermore, the mechanism of splenectomy-induced reversal of cirrhosis remains to be investigated. All these limitations should be addressed in the following study.

## 5. Conclusions

In summary, our findings provide evidence of serum markers and liver stiffness measurement suggesting that splenectomy may induce long-term reversal of cirrhosis. Further investigation of the mechanism is necessary.

## Figures and Tables

**Table 1:** Patients' demographic and clinical characteristics (*n* = 62).

Parameters	Mean ± SD or *N* (%)
Sex	
Male	34 (54.84)
Female	28 (45.16)
Age, year	49.60 ± 9.58
Hemorrhage, times	1.63 ± 1.96
Child-Pugh score	6.34 ± 1.23
Child-Pugh rank	
A	36 (58.06)
B	24 (38.71)
C	2 (3.23)
History of disease	
HBV	47 (75.81)
HCV	9 (14.52)
Alcoholic fatty liver	10 (16.13)
PBC	3 (4.84)
PSC	1 (1.61)
AIH	2 (3.23)
Surgery time (minutes)	214.25 ± 49.28
Operative bleeding (ml)	201.64 ± 187.56
Transfusion of RBC (ml)	91.80 ± 257.10
Transfusion of plasma (ml)	170.49 ± 287.14
Preoperation	
HA (*μ*g/l)	126.34 ± 93.52
LN (*μ*g/l)	62.71 ± 130.87
PIIINP (*μ*g/l)	12.14 ± 10.13
C-IV (*μ*g/l)	98.74 ± 84.31
FibroScan (kPa)	22.95 ± 15.54
Liver volume (cm^3^)	1055.88 ± 306.72

HBV: hepatitis B virus; HCV: hepatitis C virus; PBC: primary biliary cirrhosis; PSC: primary sclerosing cholangitis; AIH: autoimmune hepatitis.

**Table 2:** Change of patients' platelet counts and Child-Pugh results after splenectomy.

Parameters	Preoperation	1 day	1 week	1 month	3 months	6 months	12 months	24 months
Platelet counts	56.26 ± 37.06	91.94 ± 44.98^∗^	293.21 ± 137.10^∗^	302.90 ± 137.92^∗^	280.82 ± 133.10^∗^	273.78 ± 140.87^∗^	255.79 ± 78.79^∗^	255.57 ± 110.05^∗^
Child-Pugh								
Score	6.34 ± 1.23	—	—	5.46 ± 0.65^∗^	5.49 ± 0.87^∗^	5.38 ± 0.67^∗^	5.26 ± 0.75^∗^	5.00 ± 0.00^∗^
Class								
A	36 (58.06)	—	—	54 (91.53)	41 (91.11)	38 (95.00)	33 (97.06)	7 (100.00)
B	24 (38.71)	—	—	5 (8.47)	4 (8.89)	2 (5.00)	1 (2.94)	0
C	2 (3.23)	—	—	0	0	0	0	0

The overall changes of platelet counts and Child-Pugh results were significant among all the time points (all *P* < 0.001), and the significance compared to preoperation, ^∗^*P* < 0.05.

**Table 3:** Multivariate linear regression of serum markers with GEE models.

Parameters	HA (*μ*g/l)		LN (*μ*g/l)		PIIINP (*μ*g/l)		C-IV (*μ*g/l)	
	*β* ^1^ (95% CI)	*P*	*β* (95% CI)	*P*	*β* (95% CI)	*P*	*β* (95% CI)	*P*
Sex								
Male	ref.	—	ref.	—	ref.	—	ref.	—
Female	−14.75 (−89.27-59.78)	0.698	12.73 (−48.92-74.38)	0.686	−0.12 (−2.32-2.08)	0.915	−6.88 (−24.92-11.15)	0.455
Age, year	4.07 (0.31-7.84)	0.034	1.50 (−0.93-3.92)	0.227	0.11 (0.00-0.21)	0.045	1.05 (0.22-1.88)	0.013
Child-Pugh score	49.69 (-20.02-119.39)	0.162	34.09 (−26.09-94.26)	0.267	2.26 (0.58-3.95)	0.008	15.07 (5.17-24.97)	0.003
Time								
Preoperation	ref.	—	ref.	—	ref.	—	ref.	—
1 week	52.80 (8.43-97.17)	0.020	0.81 (−12.76-14.38)	0.907	−1.73 (−4.37-0.92)	0.201	−21.19 (−48.56-6.19)	0.129
1 month	123.87 (12.83-234.90)	0.029	−15.00 (−29.98--0.03)	0.050	−5.52 (−7.86--3.18)	<0.001	−48.54 (−70.35--26.73)	<0.001
3 months	10.17 (−22.15-42.50)	0.537	−6.89 (−23.54-9.76)	0.417	−5.60 (−8.01--3.19)	<0.001	−54.78 (−79.15--30.42)	<0.001
6 months	23.12 (−49.08-95.32)	0.530	−16.24 (−34.62-2.13)	0.083	−6.38 (−9.62--3.13)	<0.001	−63.31 (−89.55--37.08)	<0.001
12 months	41.75 (−112.45-195.95)	0.596	−1.37 (−23.61-20.86)	0.904	−7.21 (−10.17--4.25)	<0.001	−66.07 (−91.38--40.75)	<0.001

^1^Regression coefficient *β*. Abbreviations: CI: confidence interval; HA: hyaluronan; LN: laminin; PIIINP: amino-terminal propeptide of type III procollagen; C-IV: type IV collagen.

**Table 4:** Multivariate linear regression of imaging markers with GEE models.

Parameters	FibroScan (kPa)		Liver volume (cm^3^)	
	*β* ^1^ (95% CI)	*P*	*β* (95% CI)	*P*
Sex				
Male	ref.	—	ref.	—
Female	−3.09 (−8.27-2.09)	0.242	−115.41 (−251.37-20.55)	0.096
Age (years)	0.18 (−0.12-0.49)	0.235	1.11 (−4.71-6.92)	0.708
Child-Pugh score	3.94 (0.33-7.56)	0.032	−19.22 (−86.99-48.56)	0.578
Time				
Preoperation	ref.	—	ref.	—
1 month	−3.11 (−5.82--0.41)	0.024	21.07 (−16.41-58.55)	0.270
3 months	−4.07 (−6.62--1.52)	0.002	25.32 (−16.24-66.88)	0.232
6 months	−5.90 (−8.85--2.94)	<0.001	32.34 (−14.75-79.43)	0.178
12 months	−7.29 (−10.67--3.91)	<0.001	25.83 (−24.75-76.40)	0.317
24 months	−10.63 (−14.82--6.44)	<0.001	111.02 (17.87-204.18)	0.019

^1^Regression coefficient *β*. CI: confidence interval.

## Data Availability

The data used to support the findings of this study are included within the article.
